# Global Health Governance: um desafio crescente

**DOI:** 10.1590/1516-3180.2013.1315746

**Published:** 2013-10-01

**Authors:** Alessandro Wasum Mariani, Paulo Manuel Pêgo-Fernandes

**Affiliations:** I MD. Thoracic Surgeon, Instituto do Coração (InCor), Hospital das Clínicas (HC), Faculdade de Medicina da Universidade de São Paulo (FMUSP), São Paulo, Brazil.; II MD, PhD. Associate Professor, Discipline of Thoracic Surgery, Instituto do Coração (InCor), Hospital das Clínicas (HC), Faculdade de Medicina da Universidade de São Paulo(FMUSP), São Paulo, Brazil.

The concept of Global Health Governance (GHG) was created through the realization that in a globalized world, national or even regional healthcare management and solutions do not meet all the demands that are generated through the growing international traffic of people, goods and (why not?) ideas.

One recent crisis that made it clear that the current system is inadequate was the appearance of the H1N1 pandemic in 2009.^1^

There is no exact definition for the term Global Health Governance, which certainly leaves it subject to different interpretations. Nonetheless, our understanding is that GHG comprises a set of regulations, measures and systems that are created to deal with problems of international and transnational interdependence within the field of healthcare.

It is important to separate the notions of governance and government. Both refer to activities directed towards creation of rules and social systems. However, the concept of government suggests activities that are backed by formal authority, while governance ^2^

The origins of global health governance were rooted in negotiations between nations that initially done bilaterally between neighboring countries. As the world moved forward towards so-called globalization, the complexity of international health issues increased. The need for a more plural form both for the debate and for the implementation of rules culminated in 1948 with the creation of the World Health Organization (WHO).^3^

WHO is without doubt the largest institution within the scenario of global health governance, and it is the only one that has a role of authority in relation to governments, given that it is the official body of the United Nations for health issues. Its activities can be summarized in terms of providing leadership, creating and developing international rules and agreements, fostering health programs, articulating political agendas, providing technical support for nations and monitoring and evaluating health trends.^4^

In addition to current issues that are directly involved with global health governance, WHO has proposed, as a matter of priority, that there should be a debate about a kind of "universal healthcare coverage".^5^ This ambitious step towards constructing a more egalitarian world, at least with regard to health, is based on two components: guaranteed access to basic services relating to health promotion, preventive care, treatment and rehabilitation, with protection of funding, thereby avoiding excessive expenditure that might make programs unviable. Although this is considered to be an initiative of worldwide nature, development of universal healthcare coverage would have a large impact on less developed countries, which would need to readjust their mostly fragile national healthcare systems to a minimum standard.

According to Frenk and Moon,^6^ in an article published in the New England Journal of Medicine in 2013, there are basically three major obstacles to constructing a real globalized governance system:

• Each country's sovereignty: the absence of a global government compromises application and supervision of rules and systems within some nations;

• Sectorization: healthcare is a final product from the intersection of several sectors of the economy, such as the pharmaceutical industry, medical services industry, education industry, insurance industry, etc. Each of these has its own particular features;

• Responsibility: with the exception of WHO, which carries the seal of approval of the United Nations, there is great weakness due to lack of legitimacy, both among the nongovernmental organizations and among the agreements and international systemsthat account for a good proportion of present-day global health governance. This lack of legitimacy makes it difficult or perhaps impossible to attribute responsibilities to these players.

Brazil, which was ranked as an emerging power within this scenario by Fidler^7^ in 2010, cannot hold back from actively participating in constructing this GHG system, which might be as an opinion-former or even as a provider of human resources and logistics.

Lastly, although global health governance is a concept that remains unknown to the majority of the worldwide medical community, this discussion should not be left only for governments or international institutions to address. After all, today we have the chance to construct a system on foundations of greater solidity, which will have increasing impact on healthcare for future generations.
